# Induced cancer stem-like cells as a model for biological screening and discovery of agents targeting phenotypic traits of cancer stem cell

**DOI:** 10.18632/oncotarget.2356

**Published:** 2014-08-17

**Authors:** Mayuko Nishi, Hidenori Akutsu, Ayumi Kudoh, Hirokazu Kimura, Naoki Yamamoto, Akihiro Umezawa, Sam W. Lee, Akihide Ryo

**Affiliations:** ^1^ Department of Microbiology, Yokohama City University School of Medicine, Yokohama, Japan; ^2^ Department of Reproductive Biology, National Research Institute for Child Health and Development, Tokyo, Japan; ^3^ Infectious Disease Surveillance Center, National Institute of Infectious Diseases, Tokyo, Japan; ^4^ Department of Microbiology, National University of Singapore, Singapore; ^5^ Cutaneous Biology Research Center, Massachusetts General Hospital and Harvard Medical School, Charlestown, MA, USA

**Keywords:** Cancer stem cell, p21Cip1, drug screening, senescence, tumor sphere formation

## Abstract

Cancer stem cells (CSCs) retain the capacity to propagate themselves through self-renewal and to produce heterogeneous lineages of cancer cells constituting the tumor. Novel drugs that target CSCs can potentially eliminate the tumor initiating cell population therefore resulting in complete cure of the cancer. We recently established a CSC-like model using induced pluripotent stem cell (iPSC) technology to reprogram and partially differentiate human mammary epithelial MCF-10A cells. Using the induced CSC-like (iCSCL) model, we developed a phenotypic drug assay system to identify agents that inhibit the stemness and self-renewal properties of CSCs. The selectivity of the agents was assessed using three distinct assays characterized by cell viability, cellular stemness and tumor sphere formation. Using this approach, we found that withaferin A (WA), an Ayurvedic medicine constituent, was a potent inhibitor of CSC stemness leading to cellular senescence primarily via the induction of p21^Cip1^ expression. Moreover, WA exhibited strong anti-tumorigenic activity against the iCSCL. These results indicate that our iCSCL model provides an innovative high throughput platform for a simple, easy, and cost-effective method to search for novel CSC-targeting drugs. Furthermore, our current study identified WA as a putative drug candidate for abrogating the stemness and tumor initiating ability of CSCs.

## INTRODUCTION

Cancer stem cells (CSCs) are defined as transformed stem cells that possess characteristics associated with normal stem cells, specifically the ability to self-renew and to form hierarchical cancer tissues [1]. Accumulating evidence has shown that CSCs reside in various solid tumors where they function as a sub-population that plays a critical role in tumor initiation, progression, metastasis, and recurrence [2, 3]. Therefore, CSCs are considered to be a source of the tumor initiation and dissemination as well as the acquisition of malignant properties [4]. CSCs are commonly resistant to conventional anti-cancer treatments such as chemotherapeutic agents and radiation therapy resulting in treatment failure [5, 6]. In order to overcome this treatment limitation, novel therapeutic strategies should aim to eliminate CSCs which should effectively eradicate the cancer initiating cell population within tumor tissues [7].

The development of new treatment strategies that target CSCs is one of the main goals of anti-cancer therapy. However, in general, a large-scale drug screening process requires a large supply of stable and homogeneous cells to ensure reproducibility of the assay [8]. It is necessary to separate a sufficient number of CSCs from tumor tissue, to amplify the CSCs while stably maintaining them in an undifferentiated state in vitro. To date, the optimal cell culture conditions for amplifying pure CSC populations remain undefined. In fact, in vitro amplified CSCs are largely incompatible with the in vivo tumor microenvironment resulting in cell death or dormancy [9]. Moreover, cellular heterogeneity of bulk of CSC populations may reduce the fidelity and feasibility of drug screening assays [10]. Recently, several research groups attempted to establish new human CSC models [11, 12]. Currently, separation and purification of cancer stem-like cells from some cancer cell lines is the most common and easy method. Cancer stem-like cells can be enriched by collecting cells expressing CSC markers such as CD133 and CD44 [13, 14]. ALDEFLUOR™ is a non-immunological method that identifies human stem/progenitor cells based on their aldehyde dehydrogenase (ALDH) activity [15]. Interestingly, co-expression of CD133^+^ and CD44^+^ with high ALDH activity identified an enriched CSC-like population within established cancer cell lines [16]. An alternative approach for the enrichment of CSCs is the use of transformed cancer cell lines that were forced to undergo the tumor sphere formation or the epithelial-to-mesenchymal transition (EMT) [17, 18]. These cells expressed surrogate CSC markers and displayed putative tumorigenic properties in vivo, which highlights the potential suitability of this model for the discovery of compounds that can selectively target CSCs [19]. Despite the advances in modeling CSCs, the genetic variability with chromosomal instabilities in over-passaged cell lines remains a limitation that prevents consistent findings in large-scale drug screening assay. Furthermore, tumor sphere culture cannot always enrich cancer stem-like cells from cancer cell lines [20].

To overcome the aforementioned caveats, we recently developed a novel method of inducing cancer stem-like cells through the reprogramming and partial differentiation of the immortalized human mammary epithelial MCF-10A cell line which has a low genetic mutation rate [21]. These cells, termed induced cancer stem cell-like 10A (iCSCL-10A), express cancer stem markers (CD44, CD133 and ALDH1) and show much higher sphere forming ability than conventional cancer cell lines even in regular cell culture media supplemented with fetal bovine serum [22]. Furthermore, only several hundreds of iCSCL-10A cells can form hierarchically-organized tumors in immunosuppressed mice in a short period [22]. In our current study, we utilized iCSCL-10A cells to identify agents that selectively prohibit the traits of CSCs. Rather than simply identifying drugs that kill CSCs, we sought to identify compounds that interfere with the self-renewal and pluripotent properties of CSCs [23]. We found that Withaferin A (WA) was a putative anti-CSC drug candidate that altered the tumorigenic properties of CSC via the induction of cellular senescence. Our current approach can thus provide an useful option for future development of anti-CSC drugs.

## RESULTS

### Development of a drug screening assay system based on CSC properties

**Figure 1:**
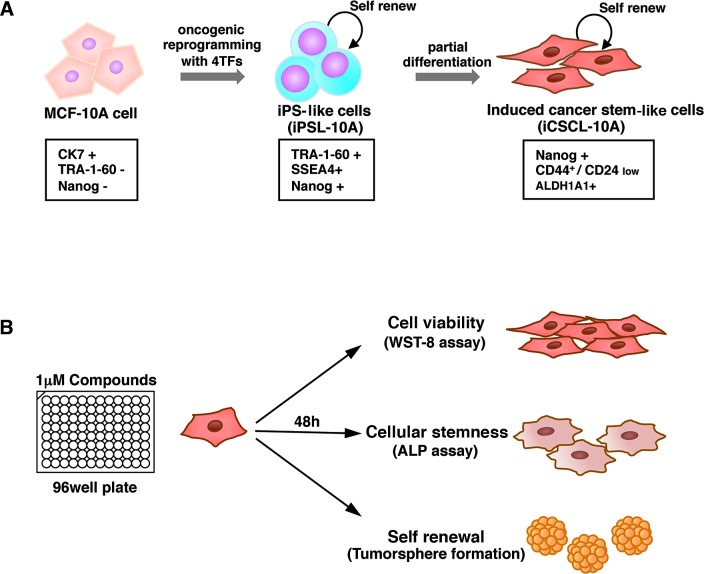
The induced CSC-like model and drug screening method (A) Non-tumorigenic MCF-10A cells were subjected to oncogenic reprogramming via the transduction of defined reprogramming factors (4TFs) to generate iPS-like cells (iPSL-10A). To generate iCSCL-10A cells with CSC properties, the iPSL-10A cells were then partially differentiated via embryoid body formation. The iPSL-10A cells acquired the ability to form hierarchically organized tumors composed of CSC-like cells and differentiated cells with multiple lineages. (B) Schematic of our drug screening method. To identify compounds with a low cytotoxicity and that abrogated CSC stemness, cell viability, cellular stemness and tumor sphere formation assays were performed in parallel.

We recently established a new CSC-like model, iCSCL-10A cells, via the introduction of defined reprogramming factors and subsequent partial cell differentiation from MCF-10A cells [22, 24]. These proliferating CSC-like cells express characteristic CSC markers, display a malignant phenotype in vitro and form tumors of multiple lineages following injection into immunocompromised mice (Figure 1A) [25]. We utilized iCSCL-10A cells for our high-throughput drug screening assay to monitor phenotypic traits of CSCs in the presence of various compounds. We aimed to identify compounds that selectively target CSCs by monitoring the specific properties of CSC, such as self-renewal and stemness, without prominent cell toxicity. The activity/selectivity of the agents was assessed using three distinct assays: 1) cell viability assay for cell toxicity, 2) alkaline phosphatase (ALP) assay for cellular stemness, and 3) tumor sphere forming assay for self-renewal. The assays were designed using the 96-well platform for large-scale drug screening (Figure 1B).

### Selection of drugs targeting CSC phenotype

**Figure 2:**
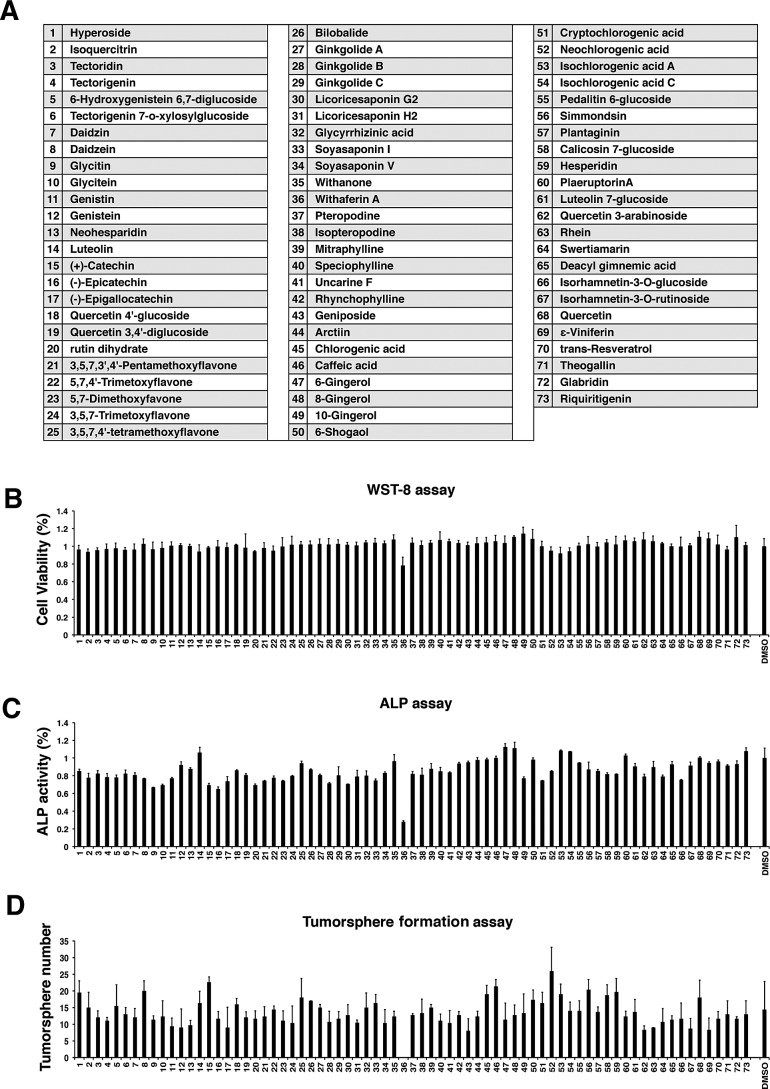
Screening of natural compounds that selectively impede the stemness of iCSCL-10A cells (A) A list of natural compounds assayed in the study. (B, C) iCSCL-10A cells (5×10^3^ cells/well in 100 μl) were plated in 96-well plates. After 24 hrs, 1 μM of compound was added to the appropriate wells. Cell viability was measured after 48 hrs using WST-8 (2-(2-methoxy-4-nitrophenyl)-3-(4-nitrophenyl)-5-(2,4-disulfophenyl)-2H-tetrazolium) activity assays (B). Alkaline Phosphatase (ALP) activity was measured after 48 hrs using the TRACP & ALP Assay Kit and a microplate reader. Values represent the mean ± SEM (n = 3) (C). (D) Quantification of tumor sphere formation. iCSCL-10A cells were plated on ultra-low attachment surface 96-well plates in the presence of 1μM of the compounds and tumor sphere formation was assessed at 48 hrs.

As an initial trial, we plated the iCSCL-10A cells in 96-well plates and tested 73 organic compounds using our three assays in parallel (Figure 2A). We used the compounds at relatively lower concentration (1 μM) in order to avoid redundant cytotoxicity. The selective toxicity against CSC-like cells was then determined using the WST-8 assay to monitor cell viability (Figure 2B). Cellular stemness was measured by monitoring expression of the stem cell marker ALP (Figure 2C). Interestingly, we found that a single compound (No. 36) drastically reduced the number of ALP-positive cells with limited cytotoxicity at 1 μM where cell viability was greater than 80%. The compound was identified as Withaferin A (WA) (Figure 2B and C). We next conducted a high-throughput tumor sphere formation assay. We designed a platform of tumor sphere formation for as short as 48 hrs to maximize the rapidity of the assay. We also noticed that WA significantly suppressed tumor sphere formation highlighting its function to abrogate the self-renewal ability of iCSCL-10A cells (Figure 2D). Based on our initial finding, WA was selected for further characterization.

**Figure 3:**
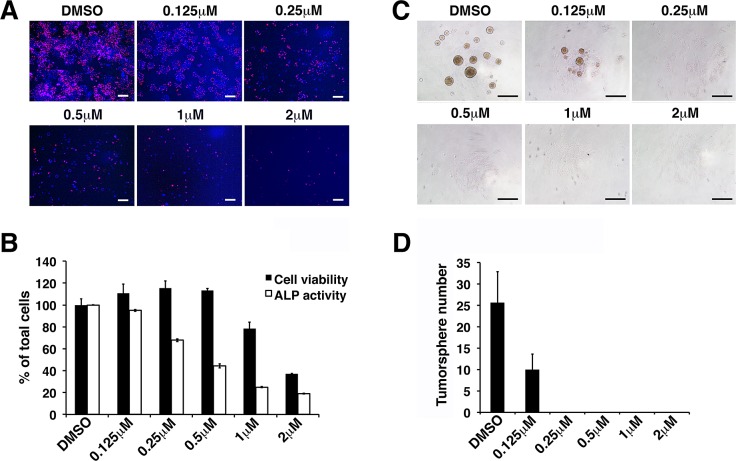
Withaferin A inhibits self-renewal and pluripotent properties of iCSCL-10A cells (A, B) iCSCL-10A cells were treated with the indicated concentrations of WA and ALP activity was measured after 48 hrs. Phase contrast images of ALP staining in iCSCL-10A cells treated with WA (A). Nuclei were counterstained with 40,6-diamidino-2-phenylindole (DAPI). Scale bar, 500 mm. Cell viability was measured after 48 hrs using WST-8 activity assays. Alkaline Phosphatase (ALP) activity was measured after 48 hrs using the TRACP & ALP Assay Kit and a microplate reader. Values represent the mean ± SEM (n = 3) (B). (C, D) iCSCL-10A cells were plated in the presence of the indicated concentrations of WA and tumor sphere formation was assessed after 48 hrs. Phase contrast images of tumor spheres (C). Values represent the mean ± SEM (n = 3) (D). Scale bar, 500 mm.

We conducted parallel experiments using varying concentrations of WA. Our results revealed that the stemness of iCSCL-10A cells was abrogated at 0.25 μM of WA as observed using the ALP assay, while the cell viability was inhibited at concentrations greater than 2μM of WA (Figure 3A, B). Tumor sphere formation was inhibited at concentrations as low as 0.125 μM (Figure 3C, D). Collectively, these results indicate that WA could selectively inhibit the stemness of iCSCL-10A cells at relatively low concentrations compared to its impact on cell viability at 10-fold to 15-fold higher concentrations. We hereafter used 1μM WA in our further studies since the effect of WA was prominent at this concentration despite of the minimum cytotoxicity.

**Figure 4:**
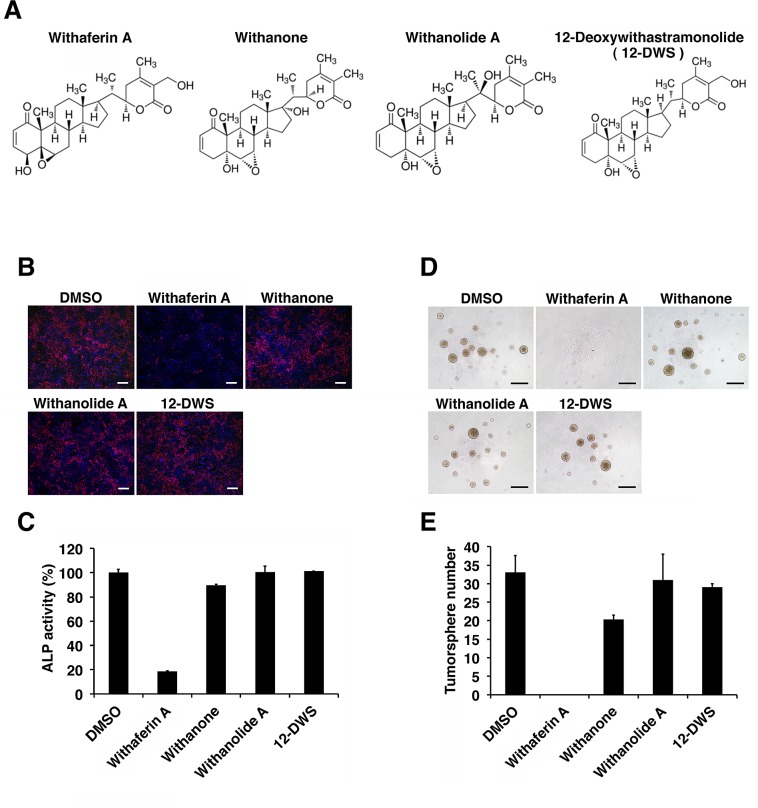
The effect of Withaferin A analogues on the expression of phenotypic CSC properties (A) Chemical structures of Withaferin A, Withanone, Withanolide A and 12-Deoxywithastramonolide (12-DWS). (B, C) iCSCL-10A cells were treated with 1 μM of Withaferin A, Withanone, Withanolide A, or 12-DWS for 48 hrs followed by the ALP assay. Phase contrast images of ALP staining (B). Values represent the mean ± SEM (n = 3) (C). Scale bar, 500 mm. (D, E) iCSCL-10A cells treated with 1 μM of Withaferin A, Withanone, Withanolide A and 12-DWS for 48 hrs and the tumor spheres were enumerated. Phase contrast images of tumor spheres are shown (D). Scale bar, 500 mm.Values represent the mean ± SEM (n = 3, E).

WA is the most abundant constituent of Withania somnifera, also known as Ashwagandha, which has been studied extensively for its biologically active constituents, steroidal lactones and withanolides [26]. To further explore the anti-CSC function of WA, we next investigated the activity of three WA compound analogues, Withanone, Withanolide A and 12-Deoxywithastramonolide (12-DWS) (Figure 4A). The parallel experiments for ALP staining and the tumor sphere assays revealed that none of the WA analogues abrogated the stemness and self-renew ability of iCSCL-10A cells (Figure 4B-E), suggesting that the anti-CSC properties were specific to WA.

### WA reduces the expression of stem cell and EMT markers

**Figure 5:**
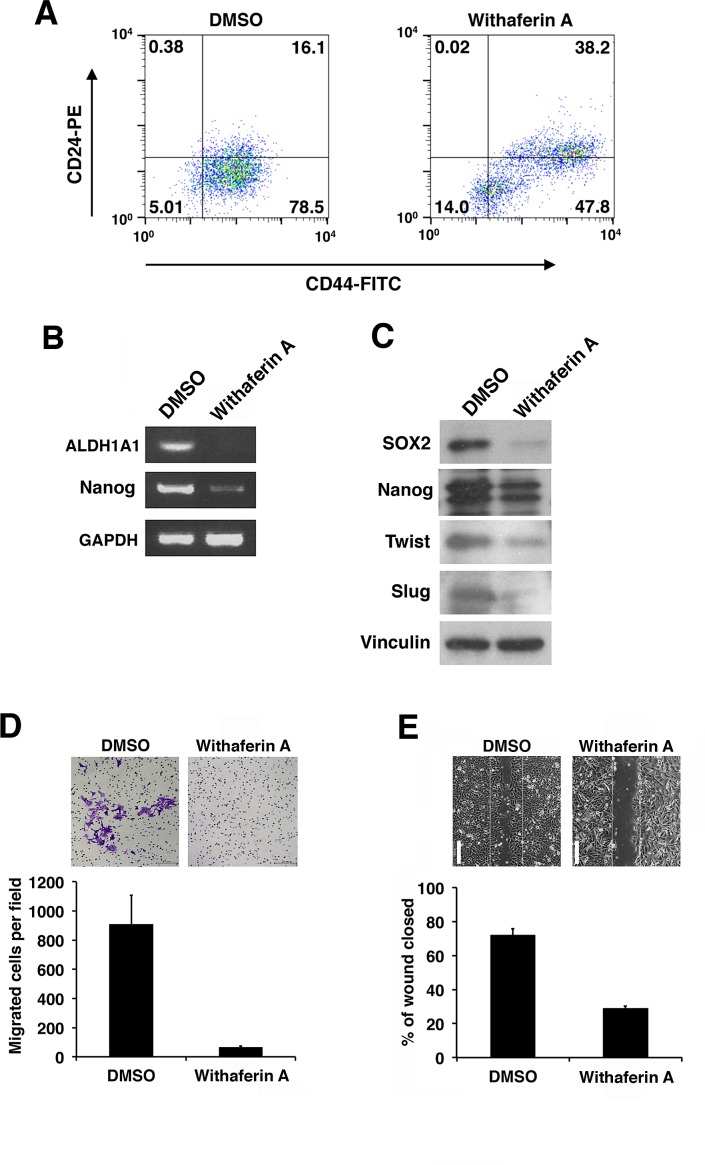
Withaferin A suppresses the CSC properties of iCSCL-10A cells (A) Flow cytometric analysis of CD44 and CD24 expression in iCSCL-10A cells treated for 48 hrs with DMSO (control) or WA (1 μM). The numbers indicate the percentage of each subpopulation according to the CD44/CD24 expression profile. (B) Semi-quantitative PCR for ALDH1A1 and Nanog in iCSCL-10A cells treated for 48 hrs with DMSO or 1μM WA. Glyceraldehyde-3-phosphate dehydrogenase (GAPDH) expression was analyzed as a control. (C) Immunoblotting of stem cell and EMT marker proteins in iCSCL-10A cells treated for 48 hrs with DMSO or 1μM WA. Vinculin was used as a loading control. (D) Cell invasion assays were performed using chemotaxis chambers in transwell tissue culture dishes as described in the Materials and methods. After 48 hrs of treatment with DMSO or 1μM WA, iCSCL-10A cells were seeded in transwells. Representative microscopic fields are shown (upper). Invasive cells were counted and transwells were scored in triplicates. The mean values ± SD were calculated from three independent experiments (lower). (E) Effects of WA on wound healing. Confluent monolayers of iCSCL-10A cells were treated for 48 hrs with DMSO or 1 μM WA and a wounded was made using a pipette tip. After 6 hrs, the cells were fixed, images were captured and wound closure was scored using ImageJ software. Phase contrast microscopy images of the cells are shown (upper). Values represent the mean ± SEM (n = 3, lower). Scale bar, 1 mm.

We further examined the effects of WA on the expression of stem cell markers on iCSCL-10A as measure of stemness. It has been well established that the CD44^+^/CD24^low^ fraction identifies the CSC population in solid tumors [13]. WA treatment significantly reduced for this fraction of iCSCL-10A cells (Figure 5A). Reverse transcription polymerase chain reaction (RT-PCR) analysis demonstrated that the expression of stem cell markers, ALDH1A1 and Nanog, were prominently reduced in iCSCL-10A cells treated with WA as compared with DMSO-treated control cells (Figure 5B). Concomitantly, WA reduced the expression of specific stem cell markers SOX2 and Nanog as measured by immunoblotting (Figure 5C). Epithelial-mesenchymal transition (EMT) is closely associated with the properties of CSCs [18]. We then assessed whether WA treatment modulated the expression of EMT regulators. WA treatment downregulated the major EMT markers, Twist and Slug (Figure 5C). Therefore, WA seems to play a dual role in iCSCL-10A cells by suppressing the expression of both stem cell and EMT markers.

### WA inhibits cell migration and invasion of iCSCL-10A

A key property of CSCs is its invasive ability. To examine the effect of WA on the invasion of CSCs, we performed a chamber invasion assay using matrigel-coated transwells. We found that WA treatment in iCSCL-10A cells resulted in a markedly reduced capacity for the cells to undergo invasion (Figure 5D).

Another important feature of CSCs is their increased mobility. To evaluate whether WA affects CSC migration in this context, wound-healing assays were performed. WA treatment significantly inhibited the wound healing ability of iCSCL-10A cells (Figure 5E). Therefore, WA treatment profoundly inhibits the tumor invasion and migration properties of CSCs.

### WA disrupts the microenvironment of CSC-like spheres

**Figure 6:**
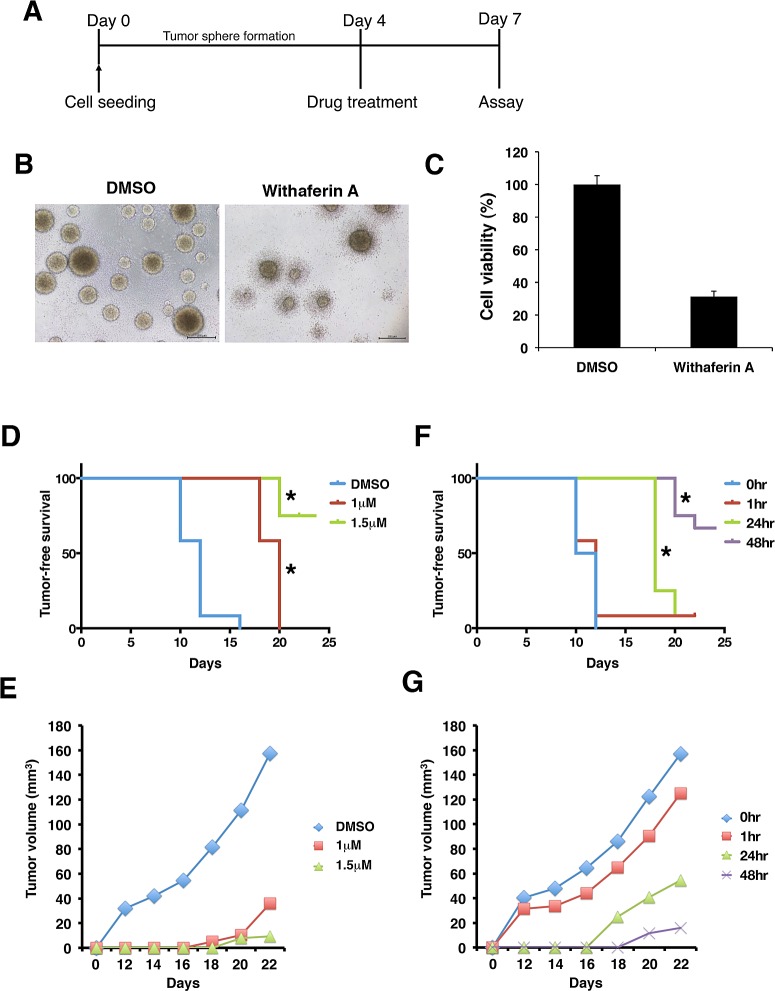
Withaferin A disrupts microenvironment and tumorigenicity of iCSCL-10A cells (A-C) Time schedule of the experiment (A). iCSCL-10A cells were dissociated and then cultures for 4 days to form spheres in ultra-low attachment surface plates followed by the treatment of WA for another 3 days. At 7 days, cell viability was monitored by CellTiter-Glo™ Luminescent Cell Viability Assay (C). Values represent the mean ± SEM (n = 3). Phase contrast images of tumor spheres (B). (D, E) iCSCL-10A cells were pre-treated with the indicated concentrations of WA for 72 hrs, and the tumor seeding ability of the cells was assessed by injecting 1×10^6^ cells into nude mice. The tumor initiation ability per injection was monitored at different concentrations. Kaplan–Meier tumor-free survival curves mice receiving each cell types after injection at day 0. Statistically significant differences were observed between DMSO v.s. 1 mM and DMSO v.s. 1.5 mM, respectively (*, P<0.01) (D). Tumor size was monitored with by external caliper at indicated time points (E). Data represent means of 12 mice per condition. (F, G) iCSCL-10A cells were treated with 1.5 μM of WA for the indicated amount of time. WA treated cells (1×10^6^ cells) were then injected subcutaneously into nude mice and the tumor initiating ability of the cells was then monitored over time. Tumor-free survival (F) and tumor size (G) was monitored as in (D) and (E). Statistically significant differences were observed between 0 hr v.s. 24 hr and 0 hr v.s. 48 hr, respectively (*, P<0.01). Data represent means of 12 mice per condition.

To examine the effect of WA on CSC microenvironment, pre-formed tumor spheres were treated with either WA or DMSO followed by morphological and cell viability analyses (Figure 6A). It was notable that WA significantly reduced the viable cell numbers forming tumor spheres. Concomitantly, tumor spheres exhibited irregular morphology with notched structure around the rims upon WA treatment while no such inhibitory effect was observed in cells treated with DMSO (Figure 6B, C). These results suggest that WA can penetrate into tumor spheres and may disrupt the microenvironment that can be important for the maintenance of CSC stemness.

### WA inhibits the tumorigenicity of iCSCL-10A

We subsequently assessed the in vivo tumor-forming ability of cells treated with WA. For these experiments, iCSCL-10A cells were treated with varying concentrations of WA in vitro for 72 hrs prior to injection into immunosuppressed mice. We observed that iCSCL-10A had a strong tumorigenic property forming tumor mass for only 10 to 12 days as shown in DMSO-treated control cells (Figure 6D). On the other hand, the pre-treatment of WA had a dose-dependent anti-tumor effect on iCSCL-10A cells in both tumor initiation and tumor size (Figure 6D, E).

Next, we assessed the optimal duration for WA pre-treatment for inhibiting tumor formation of CSCs. iCSCL-10A cells were treated with 1.5 μM of WA for varying hours prior to being injected into immunosuppressed mice. WA pre-treatment for more than 24 hrs significantly decreased both the tumor initiation and the tumor-forming ability of iCSCL-10A cells (Figure 6F, G). Taken together, these results indicate that the pre-treatment with WA can abrogate the tumorigenic properties of iCSCL-10A cells.

### WA induces the senescence of iCSCL-10A

**Figure 7:**
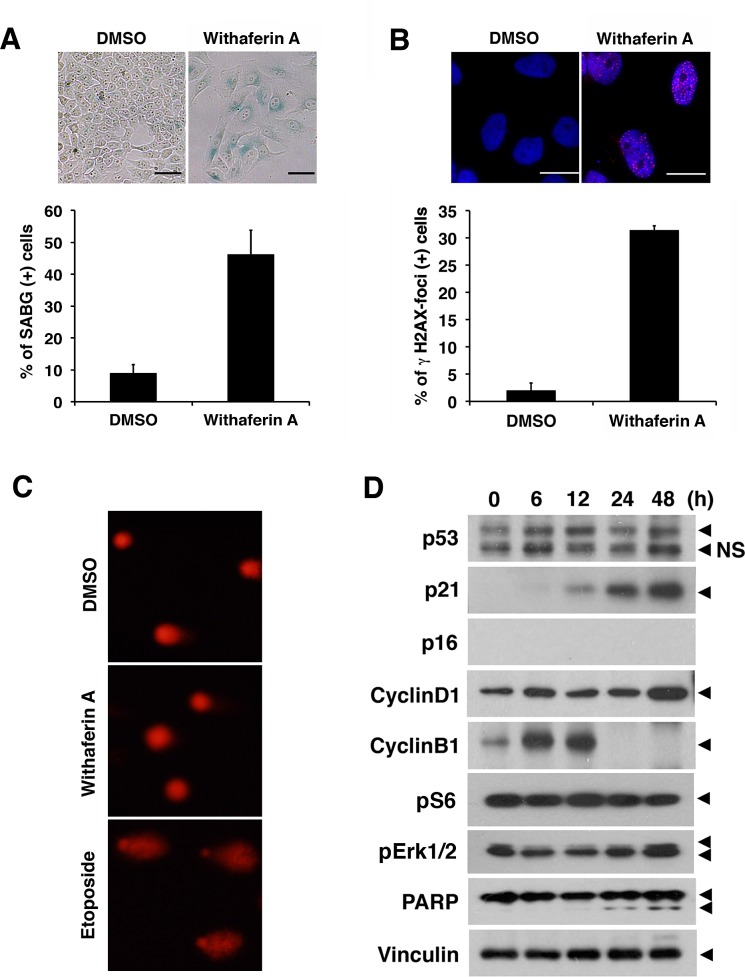
Withaferin A induces cellular senescence and increases p21^Cip1^ expression in iCSCL-10A cells (A) iCSCL-10A cells were treated with DMSO or 1μM of WA for 48 hrs and then stained for senescence-associated b-galactosidase (SABG). Phase contrast microscopy images of the cells are shown (upper). Scale bar, 100 mm. Bars indicate the percentage of SABG-positive cells for each condition and the data shown indicate mean ± SEM (n = 3, lower). One hundred cells per condition were counted and scored. (B) Immunofluorescent analysis of phospho-Histone H2A.X in iCSCL-10A cells treated with DMSO or 1μM of WA for 48 hrs. Nuclei were counterstained with DAPI. Phase contrast microscopy images of the cells are shown (upper). Scale bar, 100 mm. The number of phospho-Histone H2A.X cells was calculated and scored for three independent experiments (lower). Data shown are the mean ± SEM. One hundred cells per condition were counted and scored. (C) Cells were treated as in (B) and subjected to single-cell electrophoresis under denaturing conditions (comet assay). As a positive control, cells treated with10 μg/ml etoposide for 1 hr. (D) Immunoblotting of cell cycle related proteins in iCSCL-10A cells treated with 1 μM of WA at the indicated time points. Vinculin was used as a loading control.

We next determined the mechanism regulating WA-mediated abrogation of stemness and malignant properties of CSCs. Light microscopy revealed that cell morphology was drastically changed following WA treatment. Cells that were treated with WA were enlarged, flattened and irregular shaped, and they contained increased intracellular granules which were reminiscent of cellular senescence (Figure 7A). These cells were also positively stained with senescence-associated β-galactosidase (SABG) which was not detected in the DMSO-treated cell population (Figure 7A). To further support this observation we tested another senescence-associated marker γH2AX, a phosphorylated form of the histone variant H2AX [27]. We detected large phosphorylated H2AX foci in the WA treated cells but not in the DMSO-treated cells (Figure 7B). However, DNA damage response was not increased upon WA treatment as revealed by comet assay (Figure 7C) [28]. These results strongly indicate that the abrogation of CSC properties in iCSCL-10A cells is mediated by WA-induced senescence.

The expression pattern of cell cycle regulatory proteins was examined in WA-treated cells. There was a prominent increase in the expression of p21^Cip1^ following treatment with WA, whereas p53 protein levels remained largely unchanged (Figure 7D). p16^Ink4a^ expression was not observed due to deletion of INK4A/ARF locus in parental MCF-10A cells (Figure 7D) [29]. Cyclin B protein expression was markedly increased at the early time points but was suppressed after 24 hrs while cyclin D1 was increased at 48 hrs, which was further indicative of cellular senescence [28, 30]. It was shown that the activation of mitogenic pathways such as mammalian target of rapamycin (mTOR) or mitogen-activated protein kinase (MAPK)/mitogen-activated/extracellular signal-regulated kinase (MEK) drives geroconversion from cell cycle arrest to senescence [30, 31]. Indeed, both phosphorylated S6 ribosomal protein (pS6) as a marker of mTOR and phosphorylated extracellular signal-regulated kinase (pErk1/2), as a marker of MAPK activity, were highly expressed in WA-treated cells during the course of senescence (Figure 7D). Moreover, there was no prominent cleavage of PARP (Figure 7D), strongly suggesting that the cells underwent senescence rather than apoptosis.

### Ectopic p21^Cip1^ expression largely recapitulates WA treatment in iCSCL-10A cells

**Figure 8:**
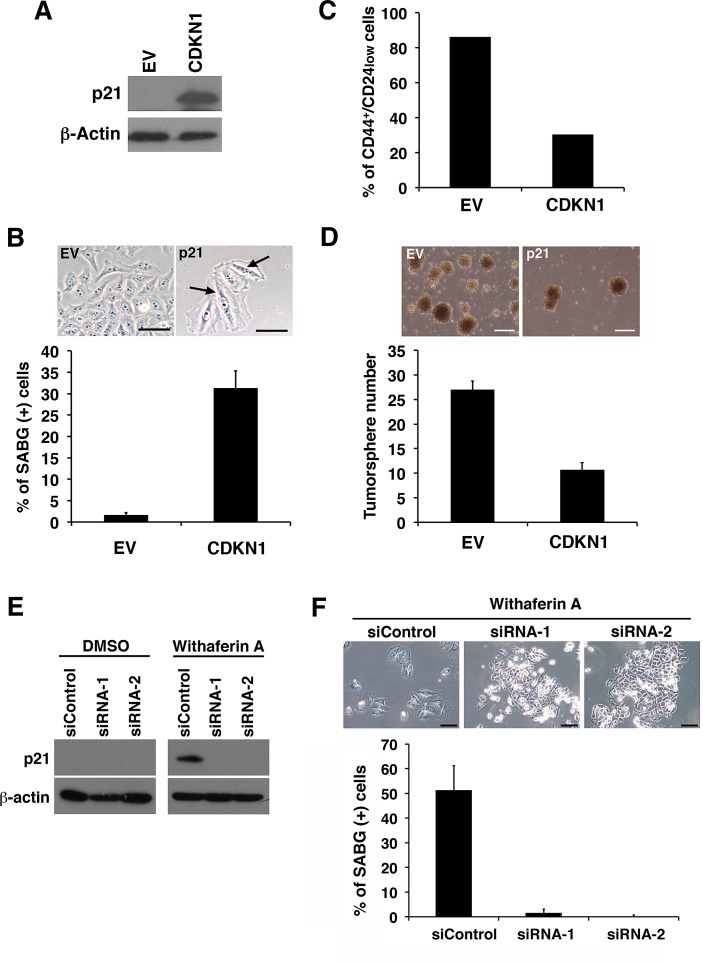
p21^Cip1^ suppresses CSC properties and induces cellular senescence of iCSCL-10A cells (A) Whole cell lysates from iCSCL-10A cells transduced with the cyclin-dependent kinase inhibitor 1 (CDKN1) encoding p21^Cip1^ or empty vector (EV) control retrovirus were subjected to immunoblotting for the expression of the indicated proteins. β-Actin was used as a loading control. (B) CDKN1-transduced iCSCL-10A cells were stained with senescence-associated β-galactosidase staining (SABG). Phase-contrast microscopy images of the cells are shown (upper). Arrows indicate positive staining shown in blue. Scale bar, 200 mm. The graph shows the percentage of SABG-positive cells for each condition (lower). Data shown indicate the mean ± SEM (n=3). (C) Flow cytometic analysis for the expression of CD44 and CD24 in iCSCL-10A cells transduced with CDKN1 or empty vector (EV). The graph shows the frequency of CD44^+^CD24^low^ cells in each of the culture conditions. (D) CDKN1 transduction abrogates the tumor sphere-forming ability of iCSCL-10A cells. Phase-contrast microscopy images of tumor spheres produced by iCSCL-10A cells transduced with CDKN1 or empty vector (EV) (upper). Data shown indicate the number of tumor spheres (means ± SEM, n=3, lower). (E, F) iCSCL-10A cells were transfected with p21^Cip1^-siRNA for 48 hrs and then treated with 1μM WA for another 48 hr. Expression of p21Cip1 was assessed by immunoblot analysis (E). β-Actin was used as a loading control. Cells were stained with senescence-associated β-galactosidase staining (SABG). Phase contrast microscopy images of the cells are shown (upper). The graph shows the percentage of SABG-positive cells for each condition (lower). Data shown indicate the mean ± SEM (n=3)(F).

In our iCSCL-10A cells, WA induced p21^Cip1^ during the course of cellular senescence. In previous studies, expression of p21^Cip1^ was tightly linked to cellular senescence [28, 32]. Therefore we evaluated whether p21^Cip1^ played a role in WA-induced senescence and resultant abrogation of CSC tumorigenicity. To test this hypothesis, iCSCL-10A cells were transduced with the cyclin-dependent kinase inhibitor 1 (CDKN1) encoding p21^Cip1^ using a retrovirus vector followed by selection with puromycin. Immunoblot analysis confirmed the stable expression of the ectopic p21^Cip1^ in CDKN1-transduced cells (Figure 8A). CDKN1-transduced cells, but not control vector transduced cells, exhibited a flattened, enlarged, and heterogeneous cell morphology which was characteristic of cells in senescence (Figure 8B). These cells also expressed SABG, which was not observed among the control vector transduced cells (Figure 8B). Concomitantly, the CD44^+^CD24^low^ fraction within the CSC population was significantly reduced in the CDKN1-transduced cells (Figure 8C). Furthermore, CDKN1 transduction significantly reduced the rate of tumor sphere formation (Figure 8D).

On the contrary, the targeted depletion of endogenous p21^Cip1^ by two different forms of siRNA prominently prohibited the WA-induced cellular senescence in iCSCL-10A cells as revealed by SABG staining in line with the suppression of WA-induced p21^Cip1^ expression (Figure 8E, F). Taken together these results indicate that the expression of p21^Cip1^ can abrogate the CSC properties of iCSCL-10A cells through the induction of cellular senescence.

## DISCUSSION

In our current study, we developed and used an in vitro CSC-like model for the identification and characterization of novel agents targeting CSCs based on phenotypic properties. Several recent studies have attempted to use cell sorting to enrich a subpopulation of cancer cell lines as a potential CSC-like model [33]. However, a major limitation of these studies is the use of cell surface markers that may or may not be restricted to CSCs, and in some cases may be improved when combined with other properties [34]. Our current approach for creating a CSC-like cell population to assay the biological functions of CSCs sought to overcome these limitations by utilizing an iPSC technology for cellular reprogramming and subsequent partial differentiation of immortalized human mammary epithelial cells [24]. Although our technique manipulates iPS-like cells with further differentiation and transformation, the cells express CSC markers and retain the ability to differentiate into multiple lineages of cancer cells following in vivo transplantation into immunosuppressed mouse [22]. Forming the self-niche, cells can be maintained in regular cell culture medium without the need for co-culturing feeder cells to maintain the stemness. Our CSC-like model system improves the ease of characterization of cancer stem cells by enabling the examination of CSC-specific functions, such as self-renewal and tumor-initiating properties.

In our current study, we utilized three different assays, cell viability, tumor sphere formation and differentiation assays. Using our approach, we directly analyzed stemness and tumorigenic properties of CSC-like cells by monitoring their phenotypic features. WA was identified as a potential anti-CSC compound using our assay system, and was further validated with subsequent biological analyses. In fact, WA abrogated the maintenance of stemness and tumorigenicity via the induction of cellular senescence. Given the selectivity of our assay, future studies may be performed to identify additional compounds that prohibit CSC properties in large-scale analyses. In this regard, our approach would be useful not only for the identification of new targets for cancer therapy but also to improve the understanding of molecular pathways involved in the maintenance of CSCs.

**Figure 9:**
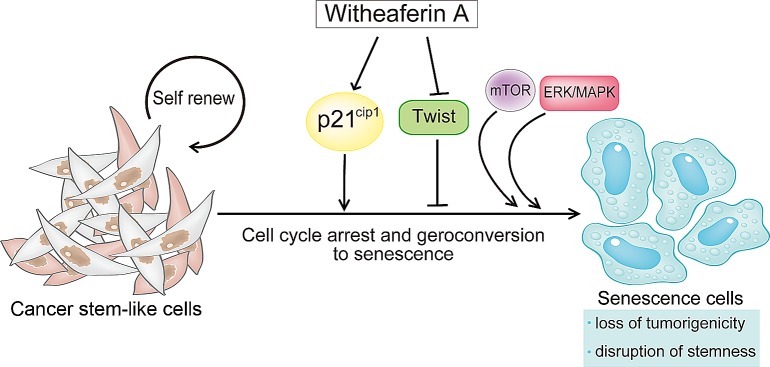
Withaferin A induces cellular senescence and prevents tumor initiating ability in CSC-like cells Schematic presentation depicting how Withaferin A (WA) can prevent CSC stemness and tumor progression. WA enhances p21^Cip1^ expression and suppresses the expression of an EMT-related transcription factor Twist. This event initiates cellular senescence program in CSC-like cells. In addition, the persistent activation of mitogenic pathways such as mTOR and/or MAPK/MEK may drive geroconversion from cell cycle arrest to senescence. Consequently, WA can abrogate CSC properties including tumor-initiating ability.

We demonstrate here that WA can abrogate the tumorigenicity of CSC-like cells. WA is a steroidal lactone that is extracted from traces in all parts of Withania somnifera except its leaves [35]. Withania somnifera is one of the most ancient herbs that is used as a medicine and a dietary supplement [26]. Several previous studies illustrated the anti-cancer activity of WA both in vitro and in vivo [36, 37]. In fact, WA has been shown to induce apoptosis of human leukemia cells via inhibition of JNK and AKT signaling as well as inhibition of NF-κB activity [38]. WA also inhibited the growth of breast cancer cells by reducing the levels of Notch family proteins in MCF-7 and MDA-MB-231 cells [39]. WA has been also shown to induce mitotic catastrophe and growth arrest in prostate cancer cells [40]. These results indicate that WA may target distinct signaling molecules for its anti-cancer activity depending on the type of cancer cell. Notably, Kim et al. recently reported that WA treatment inhibited the mammosphere formation in MCF-7 and SUM159 human breast cancer cell lines as well as mammary epithelial cells derived from MMTV-neu mice [41]. In addition to this, we here revealed a distinct function of WA to target and disrupt self-renewal pathways of CSC via inducing p21^Cip1^ and suppressing Twist thereby inducing cellular senescence program possibly via cell cycle arrest and subsequent geroconversion (Figure 9). An improved understanding of the molecular link between WA and its regulation of CSC properties as revealed by in the current study may shed new light on the molecular signature of CSCs. Moreover, the identification of WA as a novel drug that targets CSCs may validate the feasibility of our assay system for anti-CSC drug discovery.

We show here that p21^Cip1^ plays a crucial role in the abrogation of the malignant properties of CSCs via the induction of senescence. p21^Cip1^ is a potent cyclin-dependent kinase (CDK) inhibitor that functions as a downstream effector of tumor suppressors including p53 and FOXO1 [32, 42]. Recent reports indicated crucial roles of p21^Cip1^ not only in cell cycle regulation but also in the maintenance of normal and cancer stem cells. In the absence of p21^Cip1^, hematopoietic stem cell proliferation and their absolute number were increased under normal homeostatic conditions [43]. Therefore, p21^Cip1^ is regarded as a molecular switch governing the entry of stem cells into the cell cycle, and in its absence, increased cell cycling leads to stem cell exhaustion. p21^Cip1^ has been also shown to attenuate Ras- and c-Myc-dependent breast tumor epithelial-mesenchymal transition and cancer stem cell gene expression *in vivo* [44]. Our current study also uncovered that p21^Cip1^ plays a crucial role for cellular senescence in iCSCL-10A cells there by leading to the abrogation of their tumor-initiating properties. Moreover, constitutive activation of mTOR and/or MEK signaling also contributes to the geroconversion of iCSCL-10A cells toward cellular senescence [28, 30].

Activation of the senescence program in cancer cells is an attractive approach for the treatment of cancer [45]. In fact, cellular senescence has been recognized as a critical process in mammalian cells for the suppression of tumorigenesis and malignant transformation [46]. It is now clear that cellular senescence is a crucial anticancer mechanism that prevents the growth of cells at risk for neoplastic transformation into tumor initiating cells [47]. This crucial event can lead to the inhibition of metastatic dissemination, therapeutic resistance and generation of tumor cells with stem/progenitor cell properties [48, 49]. We clearly show here that WA promotes the senescence of CSC-like cells and limits their tumorigenicity and malignant characteristics. Indeed, only 48 hrs of WA treatment was sufficient to induce senescence-like morphological changes and SABG expression in iCSCL-10A cells. WA treatment increased the levels of p21^Cip1^ in iCSCL-10A cells undergoing senescence. Targeted depletion of endogenous p21^Cip1^ could block the WA-induced senescence. On the other hand, the ectopic expression of p21^Cip1^ largely recapitulated the induction of senescence and loss of CSC properties observed in WA-treated iCSCL-10A cells. These results strongly suggest that p21^Cip1^ plays a major role in inducing cellular senescence leading to the abrogation of the malignant nature in WA-treated CSCs.

WA suppressed the expression of EMT-related transcription factors including Twist. Twist plays a role in overcoming cellular senescence and in generating tumorigenic cancer stem cells [50, 51]. Indeed, Twist can abrogate oncogene-induced senescence and triggers epithelial-mesenchymal transition (EMT). Overexpression of Twist was shown to completely abrogate p16^Ink4a^ and p21^Cip1^ induction in Ras-iuduced premature senescence [52], suggesting that Twist is important for overriding cellular senescence in cooperation with oncogenes [53]. In our current study, WA strongly suppressed the expression of Twist which was in line with its induction of p21^Cip1^. EMT is a process that is closely associated with the acquisition of invasive properties in cancer progenitor or pre-cancerous cells [54]. Our current findings highlight the potential therapeutic benefits of WA treatment as a primary safe-guard system against malignant transformation, namely, the prevention of the EMT-mediated malignant conversion of pre-cancerous cells into invasive cancer stem-like cells via the activation of senescence program [55].

Herein, we developed a simple, easy, cost-effective, and highly reproducible assay system that is applicable to large-scale drug screenings. Our optimized drug screen for CSC differentiation and stemness provides excellent consistency and reproducibility for the complex biological process of CSCs. Furthermore, this drug screen can be applied to a larger number of compounds to determine more selective and effective inhibitors of CSCs. This current approach holds great promise for future development of novel drugs to eliminate CSC and hopefully provide a complete cure for tumors.

## MATERIALS AND METHODS

### Cell culture

iCSCL-10A cells were generated and maintained as described previously [22]. Cells were cultured in Dulbecco's Modified Eagle's medium (DMEM) supplemented with 10% FBS and 1% penicillin/streptomycin.

### Chemicals

Phytochemical compounds library used in this study were purchased from TOKIWA PYTOCHEMICAL Co., Ltd (Chiba, Japan). Chemicals were diluted in DMSO to 10 mM without further purification.

### Alkaline Phosphatase assay

iCSCL-10A cells (5×10^3^ cells/well) were plated in 100 ml/well in 96-well plates. After 24 hrs, 1 mM of each compound was added to the appropriate wells. Alkaline Phosphatase (ALP) activity was measured after 48 hrs using the TRACP & ALP Assay Kit (TaKaRa, Shiga, Japan). For microscopic examination, cells were stained using the VECTOR Red Alkaline Phosphatase Substrate Kit (VECTOR Laboratories, Burlingame, CA) according to the manufacturer's protocol.

### Cell Proliferation and Cytotoxicity Assays

Cell proliferation and cytotoxicity were evaluated using the Cell Counting Kit-8 (CCK-8) (Dojindo Molecular Technologies, Kumamoto, Japan). WST-8 reagent (2-(2-methoxy-4-nitrophenyl)-3-(4-nitrophenyl)-5-(2,4-disulfophenyl)-2H-tetrazolium) was added to the culture medium (1:10 dilution) and absorbance was measured at 450 nm.

### RNA isolation and RT-PCR

Total RNA was extracted using TRIzol (Life Technologies, Grand Island, NY). cDNA synthesis was performed with ReverTraAce-α (Toyobo, Osaka, Japan) in accordance with the manufacturer's instructions. Real-time PCR was performed with Premix ExTaq (Takara Bio, Shiga, Japan) using the following primers: human ALDH1A1 fwd- TAAGCATCTCCTTACAGTCAC, rev-TGTTAAGTACTTCAAGAGTCAC; human GAPDH fwd- GTGGACCTGACCTGCCGTCT, rev- GGAGGAGTGGGTGTCGCTGT; human Nanog fwd-CAGCCCTGATTCTTCCACCAGTCCC, rev-TGGAAGGTTCCCAGTCGGGTTCACC.

### Tumor sphere formation assay

Cells were seeded in 96-well ultra low-attachment surface plates (Corning) at a density of 5×10^3^ cells/well and cultured in serum-free DMEM-Ham's F12 nutrient mixture (1:1, v/v) supplemented with 5 mg/mL insulin, 0.5 mg/mL hydrocortisone, 2% B27, and epidermal growth factor (20 ng/ml).

### Cell invasion assay

Cell invasion assays were performed using 24-well transwell inserts coated with 1 mg/ml matrigel (BD Biosciences, San Diego, CA). Invasive cells in the lower chamber were counted and scored in triplicate as described previously [22].

### Wound healing assay

Cells were grown as a monolayer and a wound was made along the central axis of the plate using a pipette tip. The migration of cells into the wound was observed after 6 hrs in six randomly selected microscopic fields [56]. Wound closures were quantified using the image processing and analysis software program Image J 1.40g.

### *In vivo* tumor formation assay

Cells were washed twice with antibiotic-free and serum-free cell culture medium and resuspended in 0.1 ml of serum-free culture medium. The cell suspension was mixed with an equal volume of Matrigel (BD Bioscience, San Diego, CA) and then injected subcutaneously into 6-week-old BALB/c nude mice (CLEA Japan, Tokyo, Japan; N = 12 per condition). All animal experiments were performed under the guidelines and permission of Animal Use Protocol of Yokohama City University.

### Statistical analysis

Kaplan-Meier's method with log-rank test and Gehan-Breslow-Wilcoxon test was used to assess the differences among the samples. GraphPad Prism 6 (GraphPad Software, La Jolla, CA) was used for this purpose. A value of P < 0.01 was considered statistically significant.

### Comet assay

Comet assay was performed using CometAssay kit (Trevigen Inc., Gaithersburg, MD) according to the manufacturer's instructions.

### Immunostaining

For immunostaining, cells were fixed with 4% paraformaldehyde (PFA) for 15 min at 4°C, washed with PBS, and then permeabilized using 0.1% Triton X-100 before blocking with 5% goat serum in 0.1% BSA. Fixed cells were incubated with primary antibodies diluted in 0.1% BSA for 1 hr at room temperature followed by secondary antibody Alexa 488 or 568-conjugated anti-IgG (Life Technologies) as described previously [57].

### Antibodies

The primary antibodies used in this study were as follows: anti-phospho-H2A.X and anti-Sox2 (Millipore, Billerrica, MA, USA), anti-p53 and anti-PARP (Cell Signaling Technology, Beverly, MA), anti-p21, anti-Slug and anti-Twist (Santa Cruz Biotechnology, Dallas, TX), anti-p16 and anti-Cyclin B1 (BD Biosciences, San Diego, CA), anti-Cyclin D1 (MBL International, Nagoya, Japan), anti-Nanog (ReproCELL, Yokohama, Japan), anti-b-actin and anti-Vinculin (SIGMA-Aldrich, St. Louis, MO).
